# Reoperations after the Ross procedure: Techniques, complexity, and outcomes

**DOI:** 10.1016/j.xjse.2026.100098

**Published:** 2026-01-16

**Authors:** Elizabeth H. Stephens, Joseph A. Dearani, Nibras El Sherif, Heidi M. Connolly, Gabor Bagameri, Juan Crestanello, Alberto Pochettino

**Affiliations:** a Department of Cardiovascular Surgery, Mayo Clinic, Rochester, Minn; b Division of Pediatric Cardiology, Mayo Clinic, Rochester, Minn; c Department of Cardiovascular Diseases, Mayo Clinic, Rochester, Minn

**Keywords:** Ross procedure, reoperation, autograft, Bentall procedure

**Figure:**
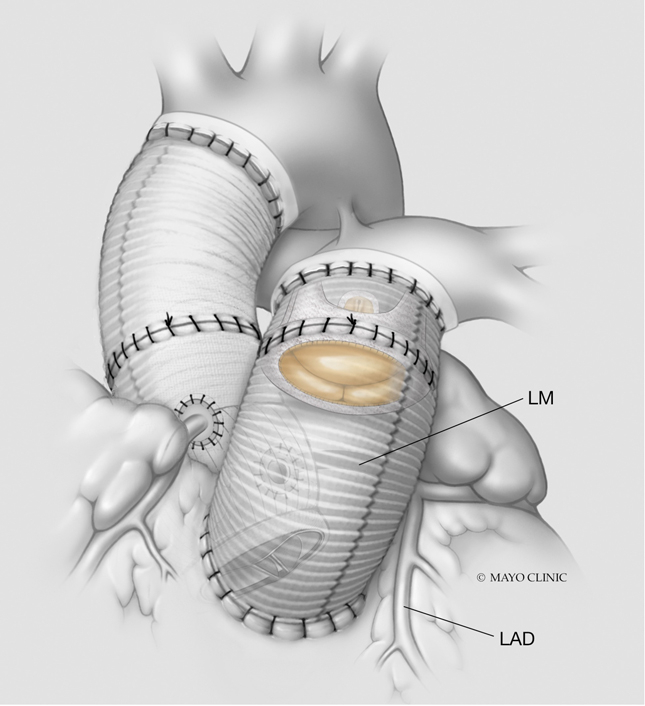
Twin-root operation after Ross. *LM*, Left main; *LAD*, left anterior descending artery.

**Central Message**

With increased interest in the Ross, ongoing surveillance, specialized surgical judgment, and further research are needed to optimize outcomes of reoperations in this population.

Although the Ross procedure has experienced renewed interest in recent years as a treatment for aortic valve pathology, the operation does require repeat interventions throughout a lifetime, of which patients and providers should be aware. This Invited Expert Opinion piece summarizes data related to reoperations after the Ross procedure and highlights technical components for these operations. Institutional review board approval and informed consent were both waived.

## Reoperations after the Ross Procedure

Relatively limited data exist to specifically examine reoperations after the Ross procedure. We reviewed our data, as a large tertiary center, for patients undergoing a reoperation after Ross procedure from 1991 to 2021,^1^ as an update from our institution's previous review.^2^ The updated cohort included 105 patients with a mean age of 27 ± 17 years at time of the Ross procedure and 37 ± 19 years at reoperation. The vast majority (84%) of Ross procedures were performed at an outside institution. Indications for the Ross procedure included bicuspid aortic valve dysfunction in 43% and congenital aortic stenosis (aortic stenosis requiring intervention in the first year of life) in 27% of patients. The Ross was the first surgical procedure for 46 of the patients (44%).

Before coming to the Mayo Clinic, patients underwent a variety of interventions, including procedures on both the left and right outflow tracts (Table 1). In most patients (64%), the primary indication for reoperation at the Mayo Clinic was autograft regurgitation, with other indications being right ventricle-pulmonary arter pathology in 17% and other indications in 19% other indications. Notably, 25% of patients were presenting for their fourth sternotomy or greater. Operations performed included autograft root operations in 78 patients (74%), most commonly root replacement in 53 patients (68%), aortic operations in 37 patients (35%), including ascending aortic replacement in 21 patients (57%) and hemiarch in 13 (35%). Right ventricular outflow tract (RVOT) interventions performed in 58 patients (53%) included pulmonary valve replacement in 37 patients (35%) and pulmonary conduit replacement in 19 patients (18%). There were a variety of other interventions (Table 2), including tricuspid valve repair in 16 (15%), tricuspid valve replacement in 2, coronary artery bypass in 8 (8%), and mitral valve interventions in 11 (11%). Despite the complexity of these operations, crossclamp time was 110 ± 83 minutes and bypass was 163 ± 90 minutes. There were 2 emergent cases after catheter interventions, one after mitral valve balloon valvuloplasty caused severe mitral regurgitation and the second after placement of a Melody transcatheter pulmonary valve caused left coronary artery occlusion.

**Table 1:** Procedures performed between the Ross procedure and reoperation

Procedures	Patient, n (%)
RV-PA conduit	9 (8.6)
Valve-sparing root replacement	4 (3.8)
AVR, RV-PA conduit, aneurysm repair	3 (2.9)
Isolated AVR	3 (2.9)
Pacemaker/ICD implantation	2 (1.9)
AVR, aneurysm repair	2 (1.9)
PVR	2 (1.9)
AVR, PVR	2 (1.9)
AVR, ascending aorta replacement	1 (0.95)
Aortic root replacement	1 (0.95)
Autograft repair, RV-PA conduit	1 (0.95)
Aneurysm repair, RV-PA conduit	1 (0.95)
Supravalvar AS repair	1 (0.95)
Total procedures	32

*RV*, Right ventricle; *PA*, pulmonary artery; *AVR*, aortic valve replacement; *ICD*, implantable cardioverter defibrillator; *PVR*, pulmonary valve replacement; *AS*, aortic stenosis.

**Table 2:** Procedures performed at reoperation

Procedures	Patient, n (%)
Pulmonary valve replacement	56 (53.3)
Bioprosthetic	28 (26.7)
Mechanical	9 (8.6)
Conduit—bioprosthetic	12 (11.4)
Conduit—mechanical	7 (6.7)
Pulmonary annular roof augmentation	15 (14.2)
RVOT reconstruction	21 (20)
Aortic valve replacement	78 (74.2)
Conduit—mechanical	37 (55.8)
Conduit—bioprosthesis	16 (15.2)
Valve—mechanical	18 (17.1)
Valve—bioprosthesis	5 (4.8)
Homograft	2 (1.9)
Aortic surgery	37 (35.2)
Isolated ascending aorta replacement	21 (57)
Combined ascending/hemiarch replacement	13 (35)
Total arch	1 (3)
Reduction aortoplasty	2 (5)
Tricuspid valve intervention	18 (17.1)
Repair	16 (88.9)
Bioprosthetic replacement	2 (11.1)
Coronary artery bypass graft	8 (7.6)
Mitral valve interventions	11 (10.5)
Repair	5 (45.4)
Mechanical replacement	4 (36.4)
Bioprosthetic replacement	2 (18.1)
ASD repair	2 (1.9)
Intraoperative RPA stent	1 (0.9)
Atrial arrhythmia ablation	2 (1.9)

*RVOT*, Right ventricular outflow tract; *ASD*, atrial septal defect; *RPA*, right pulmonary artery.

Early mortality was 5 (5%). Among the 5 early mortalities, one was a patient with endocarditis who had a recurrence postoperatively and one was a patient with endocarditis, renal dysfunction, and atrial fibrillation with 3 previous sternotomies, including mitral valve replacement who underwent aortic valve replacement, pulmonary valve replacement, and tricuspid valve repair that was complicated by acute respiratory distress syndrome and multiorgan failure. Two were multivalve cases with aortic interventions, and one was a patient who had a Ross procedure complicated by left coronary artery compression, history of radiation, pulmonary fibrosis, and portal vein thrombosis who underwent repeat coronary artery bypass, pulmonary valve replacement, and tricuspid valve repair that was complicated by acute respiratory distress syndrome and multiorgan failure.

There was a median of 6.3 years of follow-up, with late death in 14 patients (13%). Analysis showed that patients with a pre-reoperative ejection fraction <30% presented for their reoperation sooner after the Ross (6 ± 6 years vs 14 ± 6 years) than those with ejection fraction >30%. The early mortality was greater than in the recent analysis of reoperative surgery in 1183 adult congenital heart disease patients at the Mayo Clinic,^3^ and the 13% mortality over 6 years is greater than we would hope for, considering the median age of 37 years for this cohort.

The relatively high sternotomy number (>1/4 on their fourth sternotomy or greater) in this cohort may relate to morbidity, but would not be expected to directly impact early mortality. The recent review of reoperations at the Mayo Clinic found on multivariable analysis sternotomy number was not significant (*P* = .12), whereas other key factors such as age, great complexity congenital heart disease, ejection fraction, renal failure, urgent operation, and postoperative transfusion were all independently associated with mortality. Sternotomy number was, however, independently associated with composite significant morbidity/mortality, with a hazard ratio of 1.5.^3^

We recognize that our patient population and operative experience may not be broadly generalizable, given unique referral patterns. Importantly, this study does not include a true denominator and is not intended to assess the overall durability of the Ross procedure. In addition, most patients in this series underwent their original Ross operation elsewhere, and in the majority, the autograft was not reinforced, placing these patients at greater risk for autograft reintervention. Our practice is to reinforce the Ross in patients who are adult-sized.

There are other limited data focused on reoperations after the Ross procedure. The analysis by Stelzer and colleagues from 2021^4^ examined 83 patients from 1996 to 2020 with an average age of 30 years. Sixty-eight of those had autograft dysfunction, with only 3% having 3 or 4 previous operations. There were a number of operative procedures other than addressing autograft dysfunction, including 24 mitral valve operations, 14 arrhythmia operations, and 5 bypass operations. The operative mortality was 2%, and survival was 78% at 15 years after the reoperation.

Juthier and colleagues^5^ analyzed 38 patients from 1992 to 2010, undergoing reoperation after Ross which was 11% of their total Ross cohort, mean follow-up 6 years after Ross. This again was a relatively young cohort with a mean age of 35 years. Of the 38 patients, 32 patients had autograft dysfunction and 3 had endocarditis. In this study, there was no operative mortality.

Other analyses of the Ross procedure have included data on reoperation, although that was not the primary aim. These usually focused on autograft reoperation and RVOT reintervention. Such analyses can be challenging because patients do not always return to the same center for their reoperation and usually do not include concomitant procedures. Other studies have focused on autograft reoperations, such as that from Schafer's group.^6^ This included 30 patients with a median age of 41 years and median 10 years after the Ross procedure with a follow-up of 5 years. Seven underwent valve repair, 19 valve-sparing aortic root replacement, 1 valve replacement, and 3 root replacement. There were 2 perioperative deaths (7%), 2 required reoperation after repair, and freedom cardiac death was 96% in the valve repair group and 50% after replacement.

Autograft dissection can also occur requiring reoperation, and in our experience occurred in 1 patient in the series,^2^ but also has been reported by others.^7^^,^^8^ Unlike the native aortic root, in the setting of a Ross procedure, the coronary button suture lines and the distal autograft suture line protect patients from the dissection propagating into either the coronary arteries or the remaining distal aorta, but patients with an autograft remain at risk for rupture.^7^^,^^8^

In summary, patients who undergo a Ross procedure require lifelong surveillance, and some will need complex reoperations beyond the autograft and RVOT. Reoperative Ross data likely contain 2 populations: congenital aortic stenosis with or without Shone's complex—often after multiple interventions—and young adults with bicuspid aortic valve disease without additional structural abnormalities or previous interventions. Further study is needed to better define these subgroups and to understand how reoperative complexity, as well as short- and long-term outcomes, may differ between them.

## Which Operation(s) After the Ross Procedure?

Patients presenting for reoperation after a Ross require key surgical decisions. For the autograft, options include valve-sparing aortic root replacement or Bentall procedure. We consider sternotomy number, re-entry concerns, as well as right and left heart function. Many patients with autograft valve dysfunction or multiple previous operations prefer valve replacement over valve-sparing approaches. We consider valve-sparing when the cusps are structurally normal and mobile, and systolic function is normal, but tend to avoid it in hazardous re-entries that require early institution of bypass that typically results in a long bypass time. Large series have shown comparable durability of valve-sparing repair after Ross.^4^ In general, we recommend a mechanical Bentall, reserving bioprostheses for women desiring pregnancy and those with a contraindication to anticoagulation.

In general, catheter-based interventions are preferred for the RVOT, although coronary anatomy may preclude this. For patients whose primary indication is autograft dysfunction with moderate or greater right ventricle-pulmonary artery conduit dysfunction, we replace the conduit or perform a twin-root procedure at time of surgery. The conduit can also be injured during reoperation, particularly when working around the aortic root, necessitating conduit replacement. For prostheses in the RVOT, we typically favor a large, stented bioprosthesis to facilitate future valve-in-valve interventions. This can be positioned within the bed of the homograft if it is not excessively calcified or used as a conduit. In select cases—particularly in patients with multiple previous sternotomies or previous poor bioprosthetic durability—we consider a mechanical pulmonary valve to minimize need for further reoperations. These are placed as constructed conduits with anticoagulation maintained at an international normalized ratio of 2-3. Mechanical pulmonary valve replacements have shown more stable gradients over time compared with bioprostheses, although with an increased risk of bleeding (2.2% per year vs 0.1% per year).^9^ Although a “reverse Ross” has been reported with favorable outcomes, we have not yet used this technique.^10^

Patients with a previous Ross operation often require additional procedures. The goal is to address as much pathology as possible to optimize long-term outcomes while avoiding unnecessary complexity or prolonged bypass and crossclamp times. Mitral valve repair is performed for moderate or greater regurgitation, and we routinely repair the tricuspid valve when annular dilation (>4 cm) or moderate or greater regurgitation is present. Maintaining tricuspid valve competence is critical, because prosthetic pulmonary valve dysfunction is better tolerated when the tricuspid valve remains functional. In the setting of pulmonary hypertension or significant tricuspid pathology, a banded annuloplasty is performed; otherwise, a simple suture annuloplasty (de Vega) is used, adding minimal operative time. Additional procedures may also include arrhythmia surgery or management of pacing leads crossing the tricuspid valve.

### Twin-Root Procedure

**Figure 1:**
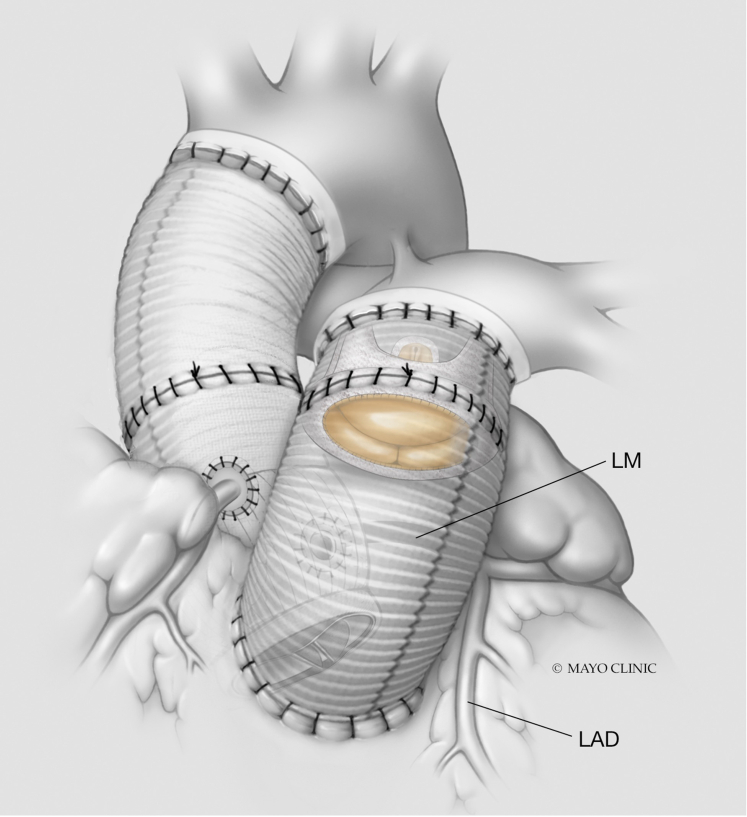
Final appearance after twin-root operation with mechanical composite aortic root replacement, hemiarch, and bioprosthetic conduit for the right ventricular outflow tract reconstruction. The pulmonary valve is placed relatively distal, distancing the ring from the left main for future catheter-based interventions and also avoiding acute angulation. *LM*, Left main; *LAD*, left anterior descending artery.

In patients with both autograft and conduit dysfunction after a Ross, we frequently perform a “twin-root” operation. The technical details are described elsewhere,^11^ but aspects are worth highlighting. The procedure is often combined with a proximal hemiarch replacement when significant distal aortic dilation is present, using a brief period of retrograde cerebral perfusion through the superior vena cava cannula. The aortic root is replaced with a valved conduit. Particular care is required when mobilizing the left coronary artery, because it is frequently adherent to the homograft. The RVOT is then reconstructed with a Hemashield graft with an interposed valve (Figure 1). Special attention is required to avoid injury to the left coronary posteriorly and the left anterior descending laterally.

## Conclusions

Reoperations after the Ross procedure are complex and often require careful management of both the autograft and the RVOT, with many patients also requiring concomitant procedures. With the resurgence of the Ross procedure, ongoing surveillance, specialized surgical judgment, and further research into patient subgroups will be essential to optimize outcomes and define the role of reoperations in this unique population.

## Conflict of Interest Statement

The authors reported no conflicts of interest.

The *Journal* policy requires editors and reviewers to disclose conflicts of interest and to decline handling or reviewing manuscripts for which they may have a conflict of interest. The editors and reviewers of this article have no conflicts of interest.
